# Cyclophosphamide for salvage therapy of chronic graft-versus-host disease: a retrospective analysis

**DOI:** 10.1007/s00277-020-04193-1

**Published:** 2020-07-26

**Authors:** Matthias A. Fante, Barbara Holler, Daniela Weber, Klemens Angstwurm, Tobias Bergler, Ernst Holler, Matthias Edinger, Wolfgang Herr, Tobias Wertheimer, Daniel Wolff

**Affiliations:** 1 Department of Hematology and Oncology, Internal Medicine III, University Hospital Regensburg, Franz-Josef-Strauß-Allee 11, 93053 Regensburg, Germany; 2 Department of Neurology, University Hospital Regensburg, Regensburg, Germany; 3 Department of Nephrology, University Hospital Regensburg, Regensburg, Germany

**Keywords:** Chronic graft versus host disease, Vasculitis-like cGvHD manifestation, Salvage therapy, Cyclophosphamide

## Abstract

We retrospectively analyzed the safety and efficacy of cyclophosphamide (cyclo) for salvage treatment of chronic graft-versus-host disease (cGvHD) and cGvHD-associated (glomerulo-)nephritis at our center between 01/2010 and 11/2019. We identified 13 patients (pts) receiving cyclo for treatment of moderate (3/13) and severe (6/13) steroid-refractory cGvHD, cGvHD-associated (glomerulo-)nephritis (3/13), or vasculitis-like CNS manifestation of cGvHD (1/13). Cyclo was started on median day 509 (range 42–8193) after cGvHD onset; the median duration of application was 153 days (range 14–486) with 2/13 currently continuing treatment. The National Institute of Health organ grading and the intensity of immunosuppression (IS) were assessed at cyclo start and repeated after 3, 6, and 12 months. Response assessment was stopped at the start of any additional new IS. The median time of follow up was 407 days (range 86–1534). Best response was 1/13 CR, 6/13 PR, 4/13 SD, 1/13 MR, and 1/13 PD (ORR 54%). Significant and durable response was observed especially in cGvHD-associated (glomerulo-)nephritis (3/3). Infectious complications > CTCAE grade III were observed in 3/12 pts. During cyclo therapy, none of the pts suffered from recurrence of underlying malignancy. Overall, cyclo was relatively well tolerated and showed responses in heavily pretreated patients but requires further evaluation within clinical trials.

## Introduction

The main benefit of allogeneic stem cell transplantation (alloHSCT) is a sustained graft-versus-malignancy effect, unfortunately often accompanied by graft-versus-host disease (GvHD) significantly contributing to non-relapse mortality and reduced quality of life [1, 2]. Chronic GvHD (cGvHD) is a multistep, host-reactive complication that occurs in up to 70% of patients [3, 4]. The underlying pathophysiology is still not fully understood, but new insights into the complex interplay of damage patterns, alterations in antigen presentation, dysregulation of B- and T-lymphocytes as well as interleukins promoting chronic inflammation, and tissue fibrosis recently advanced the field [5, 6].

Once diagnosed and staged by the current NIH consensus criteria, the standard first-line treatment of cGvHD consists of glucocorticoids with or without calcineurin inhibitors (CNIs) [7, 8]. However, about 50% of patients do not respond to first-line treatment and the second-line therapies are based mainly on phase II trials or retrospective analyses [9] and are applied off-label, except the recently FDA-approved drug ibrutinib. In the last decade, promising treatment strategies involve immunomodulating therapies (e.g., mTOR inhibitors, photopheresis) as well as *B* and *T* cell targeting agents (e.g., ruxolitinib, ibrutinib, rituximab) and further substances are under investigation [10–13].

Since its development in the late 1950s, cyclophosphamide (cyclo), a member of the nitrogen mustard family, is commonly applied in the therapy of a wide array of malignancies (including breast, lymphoid, and pediatric neoplasia) and as an essential component of conditioning regimens in bone marrow and peripheral blood stem cell transplantation (BMT/PBSCT) [14–17]. Furthermore, cyclo exerts immunosuppressive effects and is routinely used for treatment of autoimmune conditions (e.g., small vessel vasculitis, systemic lupus erythematosus) [18, 19] and prophylaxis of GvHD in the haploidentical donor transplantation setting [20, 21]. For the treatment of cGvHD, anecdotal reports were published for the use of cyclo [22, 23]. Upon oral or intravenous administration, the prodrug cyclo undergoes an enzymatical activation by the hepatic cytochrome P-450 system followed by a cytoplasmatic rearrangement process (β-elimination) to its active metabolite phosphoramide mustard causing inter- and intrastrand DNA cross-links [24].

As no data besides the anecdotal reports exist, we retrospectively analyzed the efficacy and safety of all patients receiving cyclo for treatment of cGvHD between 2010 and 2019 at the University Hospital Regensburg.

## Patients and methods

### Patients

All patients treated with cyclo for cGvHD at the transplantation program of the University Hospital Regensburg between 01/2010 and 11/2019 (*n* = 13) were included in the retrospective analysis approved by the institutional review board (no. 19-1585-104). The National Institute of Health (NIH) consensus criteria grading for cGvHD and the immunosuppressive regimen were assessed at the time of the first cyclo administration and repeated 3, 6, and 12 months of therapy as part of clinical routine during regular follow-up outpatient visits [25]. No other immunosuppressive agents were started within 4 weeks before start of cyclo and response assessment was stopped at the switch to any additional new immunosuppressive treatment line. Patients receiving cyclo as part of treatment for relapsed multiple myeloma after alloHSCT were excluded. Intention to treat was steroid-refractory cGvHD in nine patients, whereas three patients suffered from cGvHD-associated (glomerulo-)nephritis and one patient from vasculitis-like CNS manifestation of cGvHD.

### Response and event definition

Response was defined as complete remission (CR) in case of resolution of all cGvHD manifestations. Partial remission (PR) described an improvement of at least one organ grade or in case of nephritis decreased proteinuria (mg protein/g creatinine) > 50% without an increase in creatinine levels > 20%. Mixed response (MR) was defined as complete or partial remission in at least one but progression in another organ site, whereas progressive disease (PD) meant a progression without any improvements. No change in organ grading or cerebral lesions was classified as stable disease (SD).

For assessment of infectious complications, the common terminology criteria for adverse events version 5.0 (CTCAE 5.0) were applied. Toxicities with a CTCAE grades I and II were not captured in the analysis.

## Results

### Patient characteristics

We identified and analyzed 13 patients (male *n* = 8, female *n* = 5) treated with cyclo between 01/2010 and 11/2019 for cGvHD. Initial diagnoses leading to alloHSCT were myeloid disorders (acute myeloid leukemia (AML), myelodysplastic syndrome (MDS), myeloproliferative neoplasia (MPN)) in eight patients, whereas five patients suffered from lymphatic malignancies (multiple myeloma (MM), non-Hodgkin lymphoma (NHL)). All except one patient received a matched donor graft of related (*n* = 7) or unrelated (*n* = 6) donors. The predominant graft source was peripheral blood stem cells (PBSC, 12/13), while one patient received a bone marrow graft. GvHD prophylaxis consisted of a calcineurin inhibitor (ciclosporin A (CsA) or tacrolimus) and methotrexate (MTX) with (*n* = 8) or without ATG (*n* = 4). Only one patient received a prophylaxis with CsA and mycophenolate mofetil (MMF). Ten of 13 patients developed acute GvHD (aGvHD) on median day 27 (range: 13–63, no assessment in one patient) post-alloHSCT with a maximum grade (by Glucksberg criteria) of 2 or higher in 46%. Patient characteristics and history of aGvHD are summarized in Table 1.

**Table 1:** Patient characteristics

	*Value (%)*
*Patients, n (%)*	13 (100)
Male, *n* (%)	5 (39)
Female, *n* (%)	8 (61)
Age at transplantation, median (range)	53 (26–63)
Age at start of cyclo therapy, median (range)	55 (28–67)
*Diagnosis*, *n* (%)	
AML	3 (23)
MDS	2 (15)
NHL	3 (23)
Multiple myeloma	2 (15)
MPN	3 (23)
*Conditioning regime*, *n* (%)	
FBM	3 (23)
FTM	3 (23)
Treosulfan/fludarabin	3 (23)
Others	4 (31)
*Donor type*, *n* (%)	
HLA-matched unrelated	5 (38)
HLA-matched related	7 (54)
HLA-mismatched unrelated	1 (8)
*Gender mismatch*, *n* (%)	
Yes	7 (54)
No	6 (46)
*Graft source*, *n* (%)	
PBSC	12 (92)
BM	1 (8)
*GvHD prophylaxis*, *n* (%)	
ATG, CsA, MTX	8 (61)
CsA, MMF	1 (8)
CsA, MTX	3 (23)
Tacrolimus, MTX	1 (8)
*History of aGvHD*, *n* (%)	
Grade 0-I	7 (54)
Grade II-IV	6 (46)
aGvHD onset, median (range)	27 (13–63)

*n* number of patients, *AML* acute myeloid leukemia, *MDS* myelodysplastic syndrome, *NHL* non-Hodgkin lymphoma, *MPN* myeloproliferative neoplasia, *FBM* fludarabine/busulfan/melphalan, *FTM* fludarabine/treosulfan/melphalan, *HLA* human leukocyte antigen, *PBSC* peripheral blood stem cell, *BM* bone marrow, *ATG* anti-thymocyte globulin, *CsA* ciclosporin A, *MTX* methotrexate, *GvHD* graft versus host disease, *aGvHD* acute GvHD

Onset of cGvHD was quiescent in eight, de novo in three, and progressive in two patients. The onset of cGvHD was observed on median day 202 (range: 84–571) upon alloHSCT; thrombocyte count at onset was < 100/nl in five patients (no assessment available in one patient). A median of three (range 1–6) organs or sites were involved at start of treatment: eyes (6/13), lung (6/13), skin (4/13), fascia and joints (4/13), mouth (3/13), kidney (3/13), liver (1/13), genital (1/13), gastrointestinal (1/13), and CNS (1/13). Cyclo was started on median day 938 (range 218–8282) after alloHSCT and on day 509 (range 42–8193) after cGvHD onset, respectively. Prior to cyclo, a median of 2 therapy lines (0–8) had been administered. The individual patient’s treatments and the respective indication for use of cyclophosphamide are listed in Table 2. At the start of cyclo therapy, GvHD grading was moderate in three and severe in six patients. Three patients suffered from GvHD-associated (glomerulo-)nephritis with proteinuria and one patient from ischemic CNS lesions with focal neurologic deficits due to vasculitis-like cerebral cGvHD manifestation. Twelve patients received a continuous oral dose of 50 mg/day (*n* = 6) or every 2–3 days due to impaired blood counts (*n* = 6), whereas one patient was treated with a pulse therapy (initial 4 applications fortnightly, following 3 applications monthly, each 7.5 mg/kg intravenously). Steroid dose at start of cyclo treatment was in median 0.295 mg/kg (range: 0.11–1.18 mg/kg). The duration of cyclo therapy was median 153 days (range: 14–486), and therapy was still ongoing in two patients at the time of last assessment (date 11/30/2019). Median follow-up after treatment was 407 days (range: 86–1534). A more detailed overview is given in Table 3.

**Table 2:** Individual cGvHD treatments and criteria to use cyclophosphamide

patient #	Prior cGvHD therapies	cGvHD therapies at cyclo start	3 m follow-up	6 m follow-up	12 m follow-up	Criteria to use cyclo
1	Prednisolone, MMF	MMF	EOF	EOF	EOF	Steroid intolerance, high risk of relapse of myeloma (alloHSCT for refractory plasma cell leukemia)
2	Prednisolone	Prednisolone	Prednisolone	Prednisolone	NR	Progressive renal failure not responding to prednisolone
3	Ø ^(1)^	Prednisolone	Ø	Prednisolone	EOF	Systemic vasculitis with progressive renal failure, steroid intolerance
4	Prednisolone	Prednisolone	Ø	Prednisolone	Ø	Cyclo was applied for GN
5	Prednisolone, MMF	Prednisolone	Ø	Ø	EOF	Cyclo was applied for GN
6	Prednisolone, ibrutinib	Prednisolone	Prednisolone	Ø	Prednisolone	Cerebral vasculitis failing 2 lines of treatment
7	Prednisolone, everolimus, bortezomib, Bendamustine, thalidomide	Prednisolone, everolimus, thalidomide	Prednisolone everolimus	Prednisolone everolimus	Prednisolone everolimus	Concomitant relapse of multiple myeloma
8	Prednisolone, everolimus, ruxolitinib, tocilizumab, regulatory *T* cell DLI	Prednisolone, tocilizumab	EOF	EOF	EOF	Refractory cGVHD failing multiple treatment lines
9	Prednisolone, tacrolimus, ECP, MMF, everolimus, imatinib, rituximab, TNI	Prednisolone, methotrexate, tacrolimus, MMF, ECP	EOF	EOF	EOF	Refractory cGVHD failing multiple treatment lines
10	Prednisolone, everolimus	Prednisolone, everolimus	Prednisolone (*)	Prednisolone (*)	Prednisolone (*)	Steroid-refractory restrictive pulmonary allograft syndrome
11	Prednisolone, everolimus, ECP, ruxolitinib, ibrutinib, tocilizumab, regulatory *T* cell DLI	Prednisolone, tocilizumab	Prednisolone tocilizumab	Prednisolone tocilizumab	NR	Refractory cGVHD failing multiple treatment lines, history of relapse of CLL after alloHSCT
12	Prednisolone, ibrutinib, everolimus	Prednisolone	EOF	EOF	EOF	Steroid Intolerance, refractory fasciitis and restrictive pulmonary allograft syndrome
13	Prednisolone, ciclosporin A, MMF, rituximab, ruxolitinib, everolimus, abatacept	Prednisolone, MMF, everolimus	Prednisolone MMF everolimus	NR	NR	Refractory cGVHD failing multiple treatment lines

*GvHD* graft versus host disease; *3 m*/*6 m*/*12 m* 3/6/12 months; *EOF* end of follow-up; *NR* not (yet) reached; *Ø* no steroid/no immunosuppression;

*MMF* mycophenolate mofetil; *DLI* donor lymphocyte infusion; *ECP* extracorporeal photopheresis; *TNI* total nodal irradiation

*Patient started with pirfenidone 1 month after cyclo start due to lung GvHD

^(1)^Patient developed renal cGvHD manifestation during prophylactic immunosuppression with MMF (500 mg/d) and prednisolone (2.5 mg/d)

**Table 3:** Overview of cGvHD course and cyclophosphamide therapy

**patient #**	**gender**	**day of cGvHD onset**	**cGvHD type/onset**	**steroid response**	**age at cyclo start**	**day of cyclo start (after alloHSCT/** **cGvHD onset)**	**prior therapy lines**	**steroid dose at cyclo start (mg/kg bodyweight)**	**duration of cyclo therapy**	**duration of follow-up**	**organ involvement at cyclo start**	**3 m RR**	**6 m RR**	**12 m RR**	% steroid reduction (3 m/6 m)	Comment and follow-up
1	F	202	C/Q	I	46	569/367	2	–	153	1534	s (2), m (1), e (2)	–	–	–	–	New IS with ruxolitinib due to aggravated skin and mucosal GvHD after 1 month
2	M	102	O/P	D	60	238/136	1	0.87	14	274	gi (1), li (2), lu (1)	CR	CR	NR	91/96	Cyclo therapy stopped after 14 days, hospitalization with neutropenic fever (CTCAE °III)
3	F	169	C/Q	I	62	211/42	0	0.18	198	407	tubulointerstitial nephritis	PR	MR	–	100/− 200	Very good reduction of proteinuria, after 6 months new IS with MMF due to new onset of lung GvHD
4	M	571	C/N	D	66	1080/509	1	0.45	34	1507	membranous GN	PR	PR	PR	100/38	Cyclo therapy stopped after 1 month due to nausea/vomiting, persistent reduction of proteinuria
5	F	130	C/Q	D	58	549/419	2	0.12	188	751	focal segmental GN	PR	PR	–	100/100	Very good reduction of proteinuria, after 7 months new IS with ibrutinib due to increasing proteinuria
6	M	153	C/Q	NA	51	364/211	2	0.11	78	613	vasculitis-like cerebral lesions	PD	PD	PD	25/100	Stable disease for 2 months, after 3 months ocular GvHD, after 6 months new infarct areas in cMRI
7	M	421	C/N	D	53	1960/1539	5	1.18	127	359	e (3), lu (3)	SD	SD	SD	75/75	Stable diseases allowing consistent steroid reduction
8	F	162	C/Q	D	55	938/776	5	0.44	108	403	s (3), m (1), e (2), ge (1), lu (2), fas (1)	–	–	–	–	New IS with tacrolimus after 3 months due to aggravated liver and lung GvHD
9	M	258	C/N	D	37	1302/1044	8	0.47	267	1211	s (3), m (1), e (1), fas (2)	–	–	–	–	Start of MSC therapy after 1 month due to progression of cGvHD
10	M	558	C/Q	R	67	2855/2297	2	0.12	486*	486	e (1), lu (3)	SD	PD	PR	0/0	Additional antifibrotic therapy with pirfenidone after 1st follow-up
11	M	376	C/Q	D	63	1560/1184	7	0.38	155	284	s (3), fas (2)	PD	PD	NR	33/42	Progressive disease with grade 1 lung GvHD after 2 months
12	M	510	C/Q	D	28	694/184	3	0.21	22	233	lu (1), fas (1)	–	–	–	–	New IS with nintedanib due to aggravated lung GvHD
13	F	89	O/P	D	50	8282/8193	7	0.14	86*	86	e (3), lu (3)	SD	NR	–	0/−	Stable disease with clinically improved lung function, ongoing therapy
median		202			55	938/509	2	0.295	153	407					75/58	

*Ongoing therapy (2019/11/30)

Gender: *M* male, *F* female; cGvHD type/onset: *C* classical, *O* overlap, *Q* quiescent, *P* progressive, *N* de novo; steroid response: *I* intolerant, *D* dependent, *R* refractory, *NA* not applicable

Organ involvement: s skin, *m* mouth, *e* eye, *gi* gastrointestinal, *li* liver, *lu* lung, *GN* glomerulonephritis, *fas* fascia/joints, *ge* genital, *3 m/6 m/12 m RR* response rate after 3/6/12 months

*CR* complete remission, *PR* partial remission, *MR* mixed response, *SD* stable disease, *PD* progressive disease, *NR* not (yet) reached

### 3-month follow-up

The 3-month follow-up was reached by nine patients with one patient achieving complete remission and three patients partial remission resulting in an overall response rate (ORR) of 31%. Because of a sustained and progressive renal improvement for longer than 6 months, one of these patients was classified as partial remission despite a 48.8% (instead of 50%) decrease in proteinuria. In contrast, three patients had a stable disease and two further patients showed a progressive disease. New immunosuppressive medication was started in four cases precluding any response assessment. Of those, one patient received mesenchymal stem cells (MSCs) in a status of partial remission 1 month after cyclo start and three patients had aggravating cGvHD with pulmonary manifestations in two cases (Table 3). None of the patients suffered from relapse of the underlying malignancy; the failure-free survival (FFS) was 69% after 3 months. Within the first 3 months, immunosuppressants besides cyclo were reduced in four patients.

### 6- and 12-month follow-up

At 6 months after initiation of the cyclo treatment, one patient still had a complete remission and two patients showed partial remission, whereas one patient remained unresponsive. One additional patient developed progressive disease (*n* = 3) with new oral and aggravated ocular cGvHD. No further patient needed a new ISM (Table 3). After 6 months, ORR was 23% and FFS was 69%.

At 12-month follow-up, a total of six patients received new ISM and three patients have not yet reached the point of time. Two patients still showed a partial remission, one patient had a stable disease and one patient had progressive disease, displaying an ORR of 15% and an FFS of 54% (Table 3).

### Cyclophosphamide in vasculitic cGvHD manifestations

**Fig. 1:**
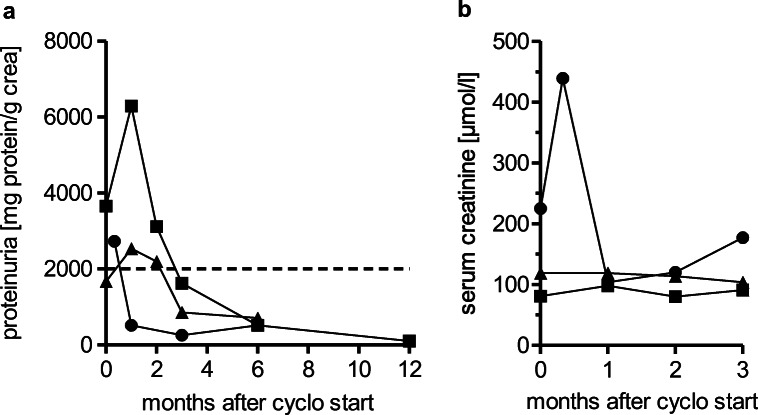
**a** Time course of proteinuria in patients III (round), IV (square), and V (triangle) after start of cyclo therapy. A distinct and durable reduction of proteinuria is shown in patients within 3 months. After 6 months follow-up is discontinued in patients III and V due to new ISM. Dashed line marks nephrotic proteinuria of 2 g/g creatinine. **b** Serum creatinine levels of patients III–V during the first 3 months of cyclo administration. In patient III, creatinine levels increase acutely within 9 days and afterward drop distinctly. Patients IV and V show a stable creatinine level

Cyclo is a commonly used treatment option in vasculitis. Therefore, we analyzed patients receiving cyclo due to cGvHD-associated vessel diseases (cGvHD-associated nephritis, cGvHD-related PML). Three patients showed a cGvHD-associated GN and nephritis, respectively, displayed by increased creatinine levels (median: 119 μmol/l, range: 81–225) and proteinuria (median: 2799 mg/g creatinine, range: 1669–3659). The subtypes were classified as membranous GN (*n* = 1) and focal segmental GN (*n* = 1) by biopsy and as tubulointerstitial nephritis by urine examination (*n* = 1). During the course of cyclo treatment, all patients showed a partial remission of proteinuria (mean of best reduction: 81.7%) with concomitantly improved renal function within 3 months after first cyclo administration (Fig. 1). Of note, one patient required new ISM due to newly developed moderate lung GvHD during treatment and one patient showed increasing proteinuria 1 month after discontinuation of cyclo therapy. All patients suffering from renal manifestation of cGvHD received angiotensin-converting enzyme (ACE) inhibitors or angiotensin II receptor blockers (ARBs), respectively. However, treatment was started before cGvHD onset due to arterial hypertension in all patients.

The patient suffering from cGvHD-associated ischemic cerebral lesions showed only a short phase of stable disease. Due to clinically significant adverse events with headache, alopecia, arthritis, and nausea, cyclo had to be discontinued after 2 months. One month later, the patient developed ocular cGvHD; 3 months later, ischemic lesions progressed and immunosuppressive therapy with methotrexate was started. Thus, we considered the patient to be a non-responder.

### Infectious complications during and after cyclophosphamide therapy

An early neutropenia was observed in two patients receiving cyclo orally after 1 and 4 weeks, respectively. Four patients were hospitalized (CTCAE grade III) due to an infection on median day 52 (range: 11–143) after start of cyclo treatment, of whom three suffered from respiratory symptoms and one from a urinary tract infection. None of the patients developed grade IV (intensive care unit) or V (death) infectious complications. The listed infections occurred during or immediately after cyclo treatment, and only one patient had meanwhile received further immunosuppressive treatment with mesenchymal stem cells. A correlation between response and infectious complications was not detectable, considering the low patient numbers.

### Steroid-sparing effect of cyclophosphamide

**Fig. 2:**
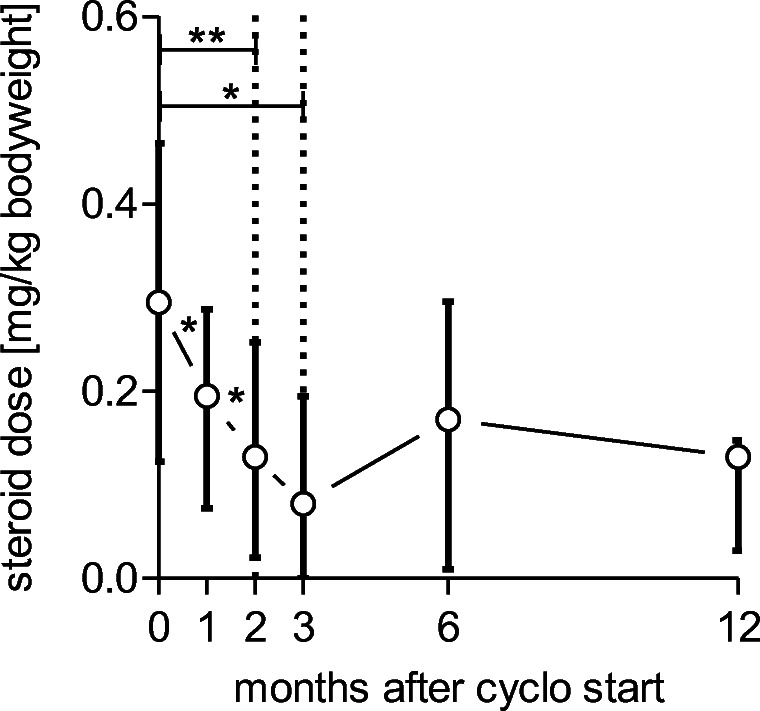
*S*teroid dose per kg bodyweight within a 12 months follow-up period. A significant reduction is observed during the first 3 months, afterwards steroid need increases. (*P* value 0.05 > * > 0.01 > ** > 0.005, treatment induced changes are analyzed with Wilcoxon test, data are presented as median with interquartile range)

As shown in Fig. 2, steroid dose of all patients reaching the follow-up was significantly reduced during the first 3 months of therapy by a median of 75% (range 0–100%). After 6 and 12 months, required steroid doses were increasing compared with the 3-month follow-up, but still reduced by 58 and 44% as compared with the initial dose. However, after 6 months, one patient received a tripled dose of steroids due to cGvHD progression, whereas all other patients had reduced or stable steroid need (Table 3).

### Predictive markers for response and safety of cyclophosphamide

Cyclo poses an uncommon cGvHD therapy and a predictive marker supported selection of suitable patients simplifies the decision-making process. Moreover, the number of patients precludes any valid statistics. Patients achieving complete or partial remission were characterized by an advanced age (mean 61.5 vs 50 years), lower number of pre-cyclo treatments (mean 1 vs 4.6), no further non-steroid ISM at cyclo start (0 vs 1.3), a shorter period until cyclo treatment (mean 281 vs 1755 days), and a lower platelet count at cGvHD onset (mean 99 vs 173/nl). However, cyclo and steroid dose did not predict response. In contrast, patients developing adverse events (CTCAE ≥ III) during or after cyclo treatment were younger (mean 44.5 vs 57.6 years), showed a higher number of pre-cyclo treatment lines (mean 4.3 vs 3.1), increased non-steroid ISM at cyclo start (mean 1.5 vs 0.7), and elevated steroid doses at cyclo start (0.69 vs 0.22 mg/kg bodyweight). In our cohort, we did not find a dependency of the cyclo dose on adverse events.

## Discussion

Steroid-refractory cGvHD poses a challenge for physicians due to the fact that empiric second-line treatments still show unsatisfactory overall responses ranging from 20 to 85% [26]. Clinical and pathophysiological similarities between cGvHD and autoimmune-mediated connective tissue diseases (e.g., systemic sclerosis, systemic lupus erythematosus) are described [27], and the application of therapeutics used in autoimmune diseases in cGvHD is an plausible strategy [28, 29]. In our single-center retrospective analysis of patients treated with cyclo, we observed a moderate ORR of 31% after 3 months. This is in fact lower as compared with the reported response of the recently FDA-approved agent ibrutinib (67%) [11]. Although cyclo represents a rather old second-line agent and has been applied mainly in prophylaxis of GvHD during the last decade, the relatively low response rate might be explained by the fact that the cohort consisted of heavily pretreated cGvHD patients having received a median of 2 prior treatment lines (range 0–8). Additionally, response to cyclo treatment substantially varied between involved organs and affected cGvHD sites.

Lung GvHD improved in 1/6 patients, while 5/6 patients showed stable disease, but three patients even developed new pulmonary involvement during therapy. Although it must be taken into account that lung involvement is mostly non-reversible and stabilization may already be a success [30], a significant proportion of patients progressed on cyclo indicating failure in a subset of patients. Cyclo has shown benefits in patients with fibrotic interstitial lung diseases [31], but can cause direct pulmonary toxicity by its active metabolites [32]. Therefore, it remains uncertain if failed response of cyclo in the context of lung GvHD is due to the underlying pathophysiology or caused by toxic side effects.

In contrast, all patients suffering from cGvHD-associated (glomerulo-)nephritis with extensive proteinuria responded after 3 months with durable improvement of renal function upon cyclo administration. This is underlined by the observation of relapsed proteinuria in one patient 4 weeks after discontinuation of cyclo therapy. Cyclo is well established for the treatment of glomerulonephritis (e.g., rapidly progressive GN, systemic lupus erythematosus associated nephritis), but most often applied as pulsed, intravenous therapy [33], which may be less well tolerated by alloHSCT patients due to hematotoxicity. As all but one patient analyzed received oral applications of cyclo, we cannot make any statements on the outcome concerning efficacy and adverse events of intravenous versus oral administration. Additionally, the oral doses varied between daily and two- to three-daily 50-mg application in our cohort. The best response (CR or PR) in the “high-dose” (daily oral or iv administration) versus “low-dose” (two- to three-daily administration) group showed similar efficiency (55 vs. 50%), however on costs of infectious complications ≥CTCAE grade III, which only occurred in the “high-dose” group. With regard to predictive marker of response to cyclo, earlier treatment lines and a shorter interval between onset of symptoms and treatment of cGvHD have been repetitively associated with higher response rates and are most likely not cyclo specific.

The major concern in patients receiving continuous treatment with cyclo is the increased risk of infectious morbidity. Our cohort showed a substantial hospitalization rate of 31% due to infectious complications (75% pneumonia, 25% lower urinary tract infection); however, no patient needed intensive care and none of the patients died in the course of the cyclo therapy. Nonetheless, these data reflect the importance of a strict selection of potential cyclo recipients and a continuous surveillance of outpatients.

Of note, we observed a meaningful reduction of steroid dose by a median of 75% and 58% (Fig. 2) after 3 and 6 months, respectively, which had been even higher if not one single patient received a tripled dose due to cGvHD progress after 6 months. Therefore, cyclo may be considered steroid-sparing agent in cGvHD therapy and be particularly suited to treat cGvHD-associated nephropathy.

In conclusion, cyclophosphamide may be an effective treatment option in some but not all cGvHD manifestations with a high response rate in GvHD-associated (glomerulo-)nephritis. To gain more experience, further evaluations in larger cohorts are necessary.
